# Natural History of Renal Neuroendocrine Neoplasms: A NET by Any Other Name?

**DOI:** 10.3389/fendo.2020.624251

**Published:** 2021-02-05

**Authors:** Andrew H. Nguyen, Michael P. O’Leary, James P. De Andrade, Philip H. G. Ituarte, Jonathan Kessler, Daneng Li, Gagandeep Singh, Sue Chang

**Affiliations:** ^1^ Department of Surgery, City of Hope, Duarte, CA, United States; ^2^ Department of Surgery, University of Iowa, Iowa City, IO, United States; ^3^ Department of Diagnostic Radiology, Division of Interventional Radiology, City of Hope, Duarte, CA, United States; ^4^ Department of Medical Oncology, City of Hope, Duarte, CA, United States; ^5^ Department of Pathology, City of Hope, Duarte, CA, United States

**Keywords:** kidney, renal, neuroendocrine neoplasm, neuroendocrine tumor, neuroendocrine carcinoma

## Abstract

Renal neuroendocrine neoplasms are rare, with descriptions of cases limited to individual reports and small series. The natural history of this group of neuroendocrine neoplasms is poorly understood. In this study, we queried the Surveillance, Epidemiology and End Results (SEER) database over a four-decade period where we identified 166 cases of primary renal neuroendocrine neoplasms. We observed a 5-year overall survival of 50%. On multivariate analysis, survival was influenced by stage, histology, and if surgery was performed. We observed that patients managed by operative management had a greater frequency of localized or regional stage disease as well as a greater frequency of neuroendocrine tumor, grade 1 histology; whereas those managed non-operatively tended to have distant disease and histologies of neuroendocrine carcinoma, NOS and small cell neuroendocrine carcinoma. This is the largest description of patients with renal neuroendocrine neoplasms. Increased survival was observed in patients with earlier stage and favorable histologies.

## Highlights

Since the first report of renal carcinoid tumor in 1966, there have been around 100 reported cases in the literature. The early reports are primarily limited to case reports and short series, with descriptions the presentation, imaging characteristics and outcomes of individual patients. More recent reports are still rare but are limited in regards to population based outcomes based on pathology. These reports typically describe these individual cases but fail to describe the overall context of each of these patients in a broader scenario by which these patients present. In our population-based study, 5-year overall survival in primary renal neuroendocrine neoplasm was 50% and was influenced by stage, histology and whether or not surgery was performed. We feel this finding will add additional knowledge to the scarce literature that is in circulation. In addition, this survey underlines the importance of developing and applying a consistent diagnostic standard, an issue that has plagued many other organ systems and cancer diagnoses.

## Introduction

Neuroendocrine neoplasms (NEN) are rare entities. As a group, their incidence today is about 7 per 100,000 individuals in the United States, with the most common primary sites being lung, gastrointestinal tract, and pancreas (1, 2). Primary renal NENs represent a poorly characterized subset of neuroendocrine neoplasms. The current medical knowledge of this disease is limited to case reports, small series, and pooled studies of reports and series (3, 4). In 2016, the 4^th^ edition of the World Health Organization (WHO) Classification of Tumors of the Urinary System and Male Genital Organs reorganized NENs into well-differentiated neuroendocrine tumors (NET), large cell neuroendocrine carcinoma (LC-NEC), small cell neuroendocrine carcinoma (SC-NEC), and pheochromocytoma (5). These categories are similar to the proposed common classification framework proposed by the International Agency for Research on Cancer (IARC) and WHO in 2018 (6).

While NENs as a group share certain histologic features such as immunohistochemical expression of chromogranin A and synaptophysin, they arise from diverse tissues where resident neuroendocrine cells play various roles depending on their location. In lung and gastrointestinal primary sites, tumors arise from native pulmonary neuroendocrine cells and a diverse group of gastrointestinal neuroendocrine cells that create and secrete bioactive products to local and distant tissues (7, 8). In the kidney, the cell of origin of these neoplasms is not well defined. There are no known native neuroendocrine cells in the renal parenchyma. Because the majority of renal NENs arise from the parenchyma, one hypothesis is that they originate from renal stem cells that develop towards neuroendocrine differentiation (3). Like neuroendocrine tumors from other sites, they have been observed on a case-by-case basis to vary in histologic grade and disease extent. Although published accounts have noted an increased incidence of renal carcinoid tumors in horseshoe kidneys (9), a larger-scale natural history and follow up outcomes of renal NENs as a group has not been attempted before.

Patients with primary NENs of the kidney may present with abdominal or flank pain, a palpable mass, weight loss, or hematuria, although a quarter of patients are asymptomatic at diagnosis (10). Patients with suspected renal NENs are typically evaluated with biochemical testing, such as urinary 5-HIAA and serum Chromogranin A, and imaging is subsequently performed for localization. Cross sectional imaging, including computed tomography (CT) of the abdomen and pelvis may demonstrate a solid, hypodense mass with mild enhancement on venous phase (11). Magnetic resonance imaging may demonstrate heterogeneous signal intensity in T1 and T2-weighted images. On renal ultrasound, the tumor may appear as a hyperechoic mass, but each of these imaging studies typically do not reveal truly distinct features to neuroendocrine tumors (12). Functional imaging with octreotide scinitigraphy of Gallium-68 DOTATATE PET/CT, may be more sensitive and specific study for well-differentiated renal NETs as has been described in NENs in other organs, although this has not been studied in renal NENs (13, 14). Nephrectomy with lymph node dissection is considered standard treatment for localized primary renal NENs. For metastatic renal NENs, long acting somatostatin analogs, tyrosine kinase inhibitors, and peptide-receptor radio nucleotide therapy that are effective in other neuroendocrine tumors are reasonable treatment options as there are no clinical trials to define optimal treatment for renal NENs at any stage (10).

In the present study, we perform the first population-based study to describe the natural history of patients with primary renal NENs. We present a series of 166 cases of primary renal NENs to study patient characteristics, tumor characteristics, and survival.

## Materials and Methods

### Patients

Patients were identified from the Surveillance, Epidemiology, and End Results (SEER) database for the years 1973 to 2014. Inclusion and exclusion criteria are depicted in **Table 1**. We identified patients by the International Classification of Disease for Oncology, morphology codes (ICD-O-3) to include 8240/3 neuroendocrine tumor, grade 1 (NET-G1); 8249/3 neuroendocrine tumor, grade 2 (NET-G2); 8246/3 neuroendocrine carcinoma, NOS (NEC-NOS); 8013/3 large cell neuroendocrine carcinoma (LC-NEC); and 8041/3 small cell neuroendocrine carcinoma (SC-NEC). Other than 8249/3 (NET-G2), these ICD-O morphology codes are the same codes used in the WHO classification. We required that the primary site of tumor to be either in the kidney (C64.9-Kidney, NOS) or the renal pelvis (C65.9-Renal pelvis). We excluded patients who did not have a histologically confirmed diagnosis and those with a prior other primary cancer. We excluded patients who were diagnosed upon autopsy or death, who were diagnosed while on hospice care or in a nursing home, and patients under the age of 18. Patient demographics included age, gender, race, environment, and year of diagnosis. Tumor characteristics included primary site, laterality, histologic type, stage, grade, and lymph node status. For the purpose of our analysis, age was converted into categorical values.

**Table 1:** Inclusion and exclusion selection criteria identifying renal neuroendocrine neoplasms.

Step	Selection Criterion	No. Remaining
1	SEER data set of patients from 1973-2014	9,675,661
2	Include cases with (1) primary site is “C64.9-Kidney, NOS” or “C65.9-Renal pelvis” AND (2) histologic type ICD-O-3 is 8013/3, 8041/3, 8240/3, 8246/3, or 8249/3	245
3	Exclude patient who did not have a histologically confirmed diagnosis	194
4	Exclude patients with prior cancer	177
5	Exclude patients diagnosed at autopsy or death	166
6	Exclude patients receiving diagnosis while in a nursing home or hospice care	166
7	Exclude cases under the age of 18	166

8240/3 (NET-G1, neuroendocrine tumor, grade 1), 8249/3 (NET-G2, neuroendocrine carcinoma, grade 2), 8246/3 (NEC-NOS, neuroendocrine carcinoma, NOS), 8013/3 (LC-NEC, large cell neuroendocrine carcinoma), 8041/3 (SC-NEC, small cell neuroendocrine carcinoma).

### Statistical Analysis

Patient demographics and tumor characteristics were summarized and compared between operatively and non-operatively managed patients. Pearson χ2 tests were used to evaluate categorical data. Age was compared across groups with a t-test. Kaplan-Meier analyses were performed to estimate 5-year overall survival (OS) and disease-specific survival (DSS). Survival times used represented time from date of diagnosis to date of death. DSS represented survival time up to death, where cause of death was identified to be due to cancer. Log-rank tests were performed to test equality among groups. A multivariate Cox proportional hazard model was performed for OS and DSS, and hazard ratios and 95% confidence intervals were reported. The Cox proportional hazards model assumptions were tested by calculating scaled Schoenfeld residuals. Analyses were performed using Stata software (StataCorp, College Station, TX). A p-value of less than 0.05 was considered statistically significant.

## Results

A total of 166 patients were identified in the SEER database with a diagnosis of primary renal NENs (**Table 2**). Eighty-five (51.2%) patients were male. The median age at the time of diagnosis was 59 years. The majority of patients were White (136 patients, 81.9%), while 14 (8.4%) were Black and 16 (9.6%) had no recorded ethnicity. Patients with renal NENs were largely identified in large urban communities (97, 59.2%), while a suburban environment (57, 34.7%) being the second most common, and only 10 (6.1%) patients were from rural environments. In the last two 6-year periods from 2003 to 2014, more patients were diagnosed with renal neuroendocrine tumors than in the time period from 1991 to 2006.

**Table 2:** Patient demographics of patients with renal neuroendocrine neoplasm diagnosed from 1991 to 2014 in the SEER database.

	All patients (n=166)
**Age**	59
**Age Groups**	
<50	48 (18.9)
≥50	118 (71.1)
**Gender**	
Male	85 (51.2)
Female	91 (48.8)
**Race/Ethnicity**	
White	136 (81.9)
Black	14 (8.4)
Other	16 (9.6)
**Community Type**	
Urban	97 (59.2)
Suburban	57 (34.7)
Rural	10 (6.1)
**Time Period**	
1991-1996	22 (13.2)
1997-2002	39 (23.5)
2003-2008	47 (28.3)
2009-2014	58 (34.9)

We then looked at tumor characteristics (**Table 3**). Seventy patients (42.2%) had documented distant disease, 56 (33.7%) had regional disease, 32 (19.3%) had local disease, and 8 (4.8%) patients had no documented stage. There were slightly more patients with right sided tumors (89, 53.6%) compared to left sided tumors (68, 40.9%). A total of 154 (92.8%) tumors were found in the renal parenchyma and 12 (7.2%) in the renal pelvis. Of histologic types as categorized by SEER, the most common were NET-G1 (56, 33.7%) and SC-NEC (55, 33.1%). There were 51 (30.7%) cases categorized as NEC-NOS. Only two cases each of NET-G2 and LC-NEC were recorded.

**Table 3:** Renal neuroendocrine neoplasm stage and tumor characteristics.

	All patients (n=166)
**Stage**	
Unknown	8 (4.8)
Local	32 (19.3)
Regional	56 (33.7)
Distant	70 (42.2)
**Laterality**	
Right	89 (53.6)
Left	68 (40.9)
Undocumented	9 (5.4)
**Location**	
Renal Parenchyma	154 (92.8)
Renal Pelvis	12 (7.2)
**Histologic type**	
Neuroendocrine tumor, grade 1 (NET-G1)	56 (33.7)
Neuroendocrine tumor, grade 2 (NET-G2)	2 (1.2)
Neuroendocrine carcinoma, NOS (NEC-NOS)	51 (30.7)
Large cell neuroendocrine carcinoma (LC-NEC)	2 (1.2)
Small cell neuroendocrine carcinoma (SC-NEC)	55 (33.1)
**Differentiation**	
Well differentiated	15 (9.0)
Moderately differentiated	15 (9.0)
Poorly differentiated	17 (10.2)
Undifferentiated/anaplastic	23 (13.8)
Undocumented	96 (57.8)
**Tumor size**	
≤2 cm	7 (4.2)
>2 cm and ≤4 cm	21 (12.6)
>4 cm	97 (58.4)
Undocumented	41 (24.7)

From the data available, only 70 of 166 patients had a reported differentiation, with a relatively even distribution of well-differentiated (9.0%), moderately-differentiated (9.0%) and poorly differentiated (10.2%) tumors. Twenty-three (13.8%) tumors were classified as undifferentiated/anaplastic. For the majority of cases in the SEER database (96, 57.8%), there was no recorded tumor differentiation. Most of the tumors were of >4 cm in size. For 24.7% of patients, primary tumor size was not recorded in the database.

**Figure 1:**
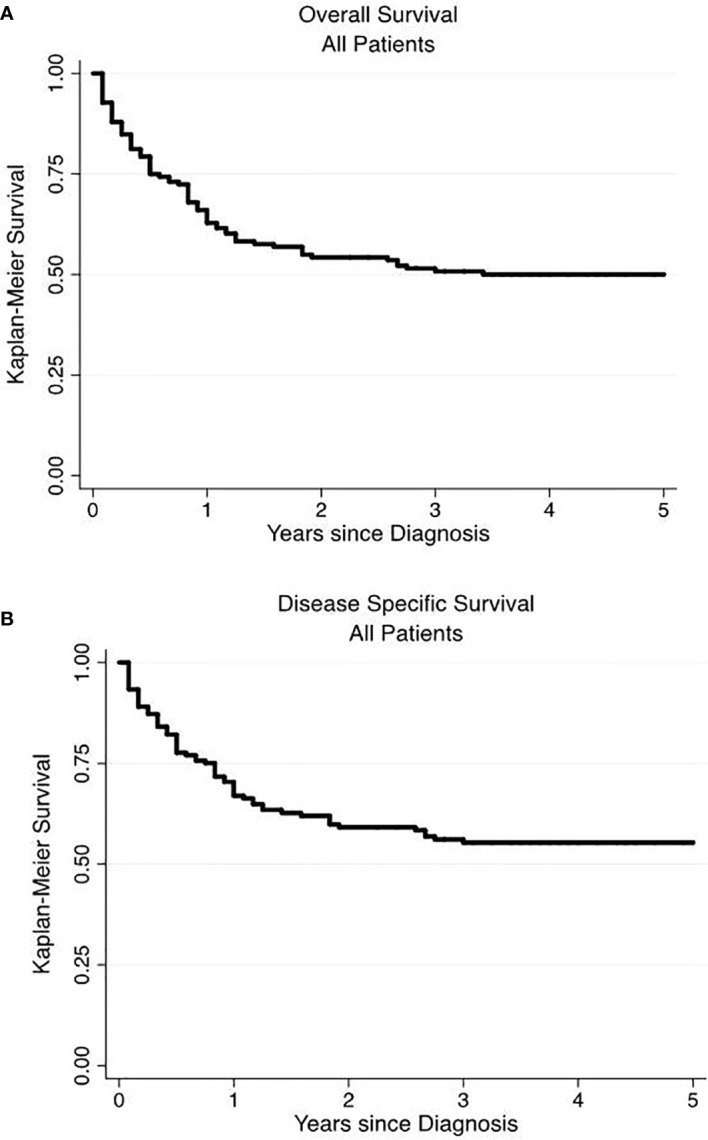
Overall and disease-specific survival of primary renal neuroendocrine neoplasms. Overall **(A)** and disease-specific **(B)** 5-year survival were 50 and 52%, respectively.

We performed a Kaplan-Meier survivor analysis of all 166 patients, observing a 5-year OS of 50% (**Figure 1A**) and a 5-year DSS of 52% (**Figure 1B**). We then performed a univariate analysis to determine how various factors contributed to overall and disease-specific survival. On univariate analysis, operative management appeared to decrease risk of all-cause mortality (HR 0.22, 95% CI 0.14–0.33) and disease specific mortality (HR 0.19, 95% CI 0.12–0.32) (**Table 4**). Older age, male gender, regional and distant disease, and histology other than NET-G1 were associated with poorer overall survival. In examining disease specific survival, of the listed risk factors, only male gender was no longer associated with poorer survival and White race was associated with increased risk. Large and small cell NECs were associated with the poorest OS and DSS.

**Table 4:** Univariate analysis of mortality (overall survival and disease-specific survival).

Risk factor	Univariate HR OS	95% CI	p-value	Univariate HR DSS	95% CI	p-value
**Age**						
<50	Ref			Ref		
≥50	1.92	1.18–3.12	0.009	2.26	1.28–3.98	0.005
**Sex**						
Female	Ref			Ref		
Male	1.62	1.08–2.43	0.02	1.46	0.94–2.29	0.094
**Race/ethnicity**						
Non-White	Ref			Ref		
White	1.79	1.01–3.18	0.043	2.04	1.05–3.98	0.035
**Year diagnosed**						
1991–1996	Ref			Ref		
1997–2002	0.73	0.41–1.29	0.279	0.74	0.39–1.39	0.348
2003–2008	0.52	0.28–0.95	0.033	0.55	0.28–1.07	0.077
2009–2014	0.4	0.21–0.76	0.005	0.4	0.19–0.81	0.012
**Stage**						
Localized	Ref			Ref		
Regional	3.16	1.43–7.02	0.005	2.77	1.09–7.04	0.032
Distant	9.9	4.54–21.59	<0.001	10.47	4.31–25.43	<0.001
Unknown	2.44	0.73–8.13	0.145	3.31	0.93–11.7	0.064
**Surgery performed**						
No	Ref			Ref		
Yes	0.22	0.14–0.33	<0.001	0.19	0.12–0.32	<0.001
**Histology**						
NET-G1	Ref			Ref		
NET-G2	6.17	0.80–47.36	0.08	7.02	0.90–54.8	0.063
NEC-NOS	3.1	1.67–5.76	<0.001	3.08	1.54–6.14	0.001
LC-NEC	11.3	2.55–50.11	0.001	13.26	2.92–60.15	0.001
SC-NEC	7.66	4.24–13.83	<0.001	7.48	3.86–14.4	<0.001

We then performed a multivariate analysis using a Cox proportional hazards model (**Table 5**). We included the same patient and tumor characteristics evaluated in the univariate analysis. We found that on both univariate and multivariate analysis, older age, male gender, White race, regional and distant disease, and histology of SC-NEC were associated with increased all-cause and disease-specific mortality. On multivariate analysis, operative management was no longer associated with statistically significant decreased risk of all-cause and disease specific mortality.

**Table 5:** Multivariate analysis of mortality (overall survival and disease-specific survival).

Risk factor	Multivariate HR OS	95% CI	p-value	Multivariate HR DSS	95% CI	p-value
**Age**						
<50	Ref			Ref		
≥50	1.25	0.73–2.12	0.418	1.41	0.76–2.63	0.271
**Sex**						
Female	Ref			Ref		
Male	1.35	0.87–2.09	0.181	1.18	0.73–1.93	0.487
**Race/ethnicity**						
Non-White	Ref			Ref		
White	1.84	0.99–3.41	0.054	2.15	1.03–4.49	0.041
**Year diagnosed**						
1991–1996	Ref			Ref		
1997–2002	1.77	0.92–3.41	0.087	1.81	0.86–3.78	0.117
2003–2008	0.95	0.47–1.89	0.879	0.92	0.42–1.99	0.839
2009–2014	0.52	0.26–1.06	0.074	0.49	0.23–1.10	0.085
**Stage**						
Localized	Ref			Ref		
Regional	2.28	0.98–5.34	0.057	1.83	0.68–4.94	0.231
Distant	4.51	1.86–10.92	0.001	4.39	1.61–11.96	0.004
Unknown	0.99	0.27–3.55	0.985	1.33	0.34–5.18	0.678
**Surgery performed**						
No	Ref			Ref		
Yes	0.28	0.16–0.48	<0.001	0.27	0.15–0.50	<0.001
**Histology**						
NET-G1	Ref			Ref		
NET-G2	9.43	1.08–91.85	0.042	10.52	1.15–96.05	0.037
NEC-NOS	2.35	1.19–4.59	0.013	2.23	1.04–4.72	0.037
LC-NEC	5.87	1.12–30.67	0.036	6.63	1.19–36.69	0.03
SC-NEC	7.22	3.56–14.65	<0.001	6.84	3.08–15.18	<0.001

To better understand differences in patients managed with operative versus non-operative management, we compared patient and tumor characteristic among these two groups (**Table 6**). We found that operatively and non-operatively managed patients were similar in patient characteristics. There were more patients with distant disease who were managed non-operatively (76.7%) than with localized (3.3%) or regional (11.7%) stage, whereas operatively managed patients had greater proportions of local (28.3%) or regional (46.7%) stage disease (**Table 7**). Histologic types of SC-NEC and NEC-NOS were more frequent among non-operative patients than operative patients.

**Table 6:** Comparing patient demographics of operative versus non-operative management.

	Non-operative (n=60)	Operative (n=106)	p-value
**Age group**			
<50	12 (20.0)	36 (34.0)	0.057
≥50	48 (80.0)	70 (66.0)	
**Sex**			
Female	27 (45.0)	54 (50.9)	0.462
Male	33 (55.0)	52 (49.1)	
**Marital status**			
Married	33 (55.0)	61 (57.5)	0.928
Not Married	25 (41.7)	41 (38.6)	
Unknown	2 (3.3)	4 (3.7)	
**Race/Ethnicity**			
Non-white	10 (16.7)	20 (18.9)	0.723
White	50 (83.3)	86 (81.1)	
**Environment**			
Urban	34 (56.7)	64 (60.4)	0.423
Suburban	20 (33.3)	37(34.9)	
Rural	6 (10.0)	11 (4.7)	
**Year period**			
1991–1996	10 (16.7)	12 (11.3)	0.755
1997–2002	14 (23.3)	25 (23.6)	
2003–2008	15 (25.0)	32 (30.2)	
2009–2014	21 (35.0)	37 (34.9)	

**Table 7:** Comparing tumor characteristics of operative versus non-operative management.

	Non-operative (n=60)	Operative (n=106)	p-value
**Stage**			
Localized	2 (3.3)	30 (28.3)	<0.001
Regional	7 (11.7)	49 (46.2)	
Distant	46 (76.7)	24 (22.7)	
Unknown	5 (8.3)	3 (2.8)	
**Laterality**			
Left	24 (40.0)	44 (41.5)	0.026
Right	29 (48.3)	60 (56.6)	
Unspecified	7 (11.7)	2 (1.9)	
**Histology**			
NET-G1	8 (13.3)	48 (45.3)	<0.001
NET-G2	1 (1.7)	1 (0.9)	
NEC-NOS	21 (35.0)	30 (28.3)	
LC-NEC	2 (3.3)	0 (0.0)	
SC-NEC	28 (46.7)	27 (25.5)	
**Location**			
Renal parenchyma	57 (95.0)	97 (91.5)	0.404
Renal pelvis	3 (5.0)	9 (8.5)	
**Cause of Death**			
Alive	7 (11.7)	62 (58.5)	<0.001
Attributed to primary renal neuroendocrine tumor	26 (43.3)	24 (22.6)	
Other cancer	11 (18.3)	6 (5.6)	
Cardiac and cerebrovascular	1 (1.6)	2 (1.9)	
Pulmonary	8 (13.3)	1 (0.9)	
Other causes	7 (11.7)	11 (10.4)	

**Figure 2:**
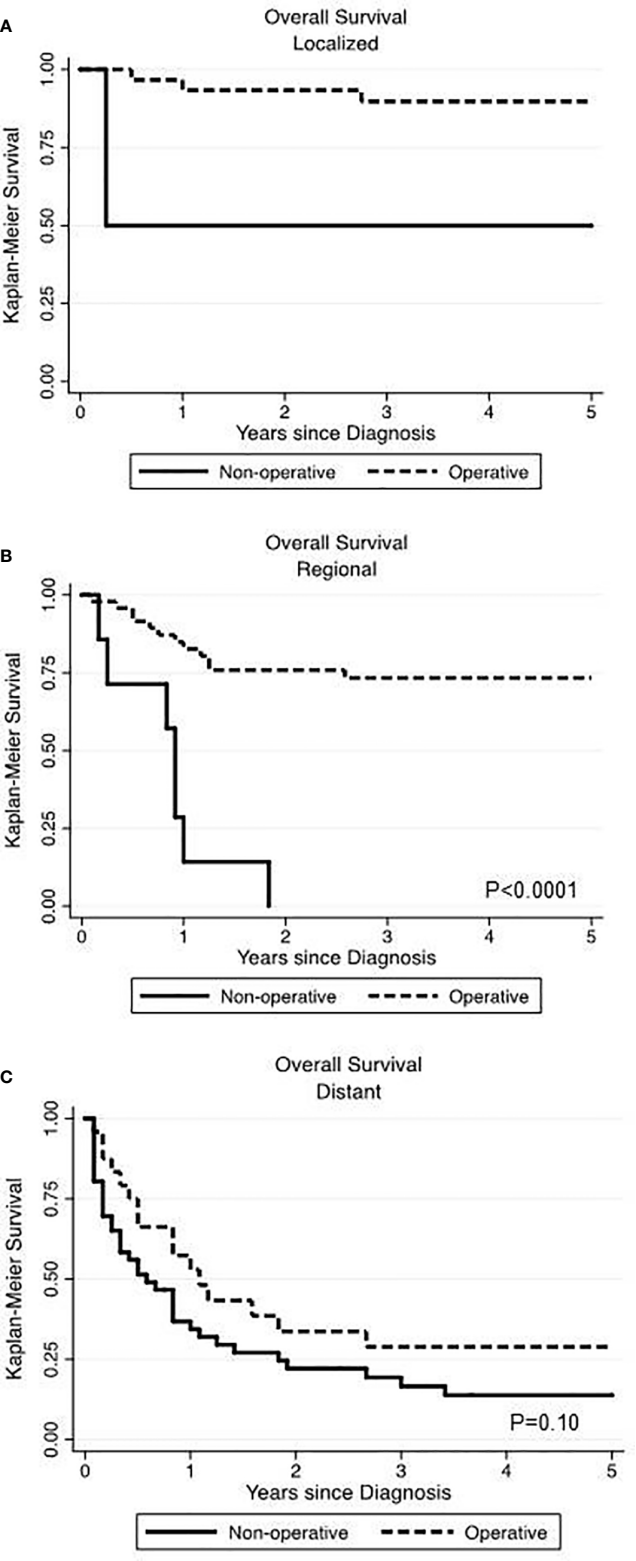
Comparing the impact of operative versus non-operative management on overall survival at various stages of primary renal neuroendocrine neoplasms. Surgery was associated with significantly improved overall survival in localized **(A)** and regional **(B)** disease but not in the setting of distant disease **(C)**.

In our data set, stage described as local, regional and distant disease was the best surrogate measure of extent of disease. We performed Kaplan-Meier analyses, assessing survival among operatively and non-operatively managed patients (**Figure 2**). In patients with local disease, all but two patients underwent operative management and those who did not have surgery eventually died from unrelated pulmonary disease (**Figure 2A**). In patients with regional disease, those managed operatively had a significant advantage in OS (p<0.0001) (**Figure 2B**). Seven patients who did not receive surgery had much poorer survival than those who underwent an operation. In those patients with distant disease, 24 patients were operatively managed and 46 patients were non-operatively managed and there was no significant difference in overall survival (p=0.10) (**Figure 2C**).

**Figure 3:**
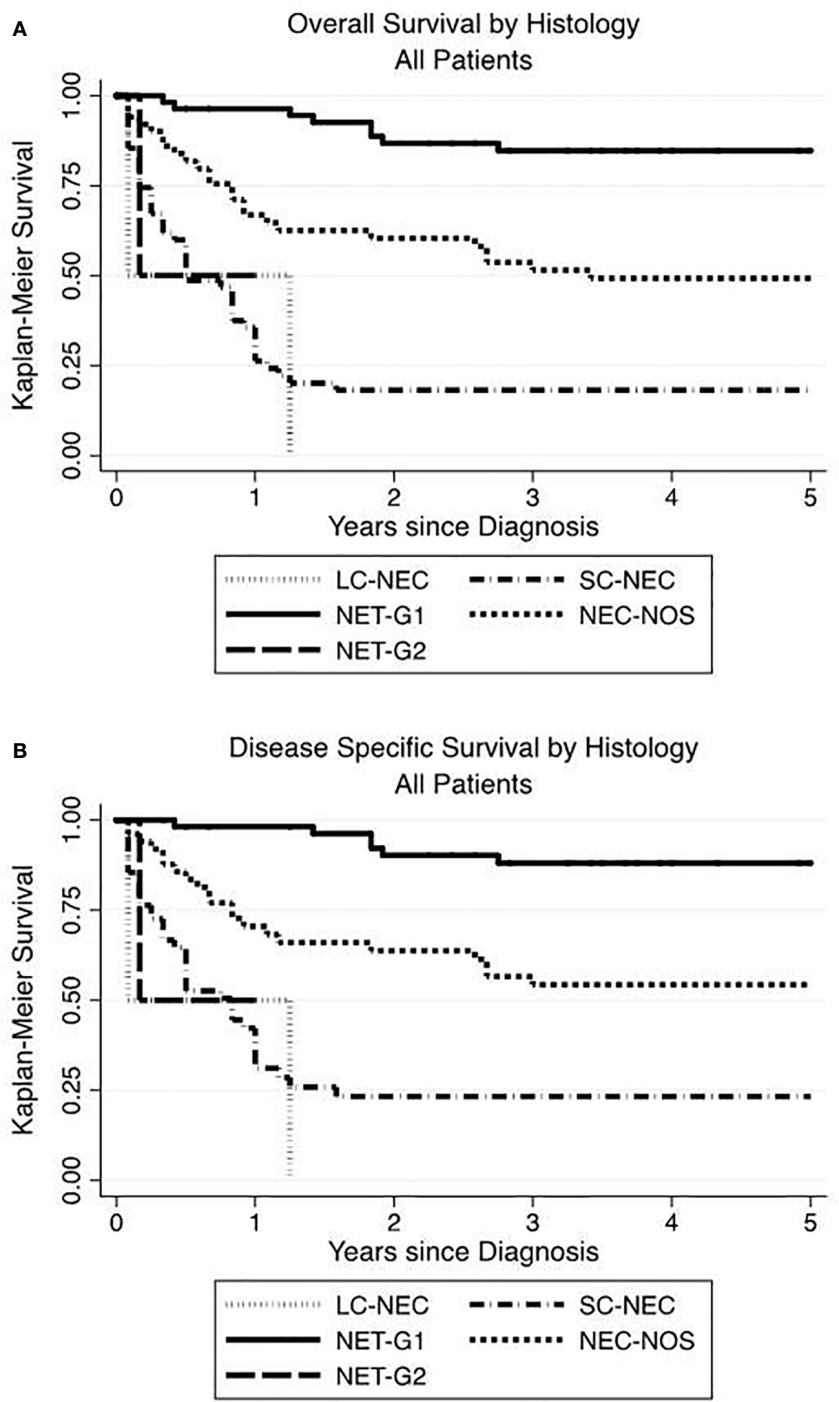
Renal neuroendocrine neoplasm histology influences overall and disease-specific survival. The highest OS **(A)** and DSS **(B)** advantage were seen in low grade NET-G1 and the poorest histologic prognosticator was with small cell neuroendocrine carcinoma.

When sorted by tumor histology, the highest OS and DSS were seen in NET-G1 and the lowest in SC-NEC. Those with NEC-NOS showed OS and DSS intermediate to NET-G1 and SC-NEC (**Figures 3A, B**.

## Discussion

As a group, neuroendocrine neoplasms are rare. There has been an increasing incidence and prevalence with more frequent detection of early stage disease and improved survival over recent decades (2). Primary renal NENs represent a minority among all NENs. The cumulative knowledge of these rare tumors is composed of case reports and series. This study represents the first population-based investigation of this rare neoplasm.

By querying the SEER database from 1973 to 2014, we identified 166 patients with primary renal NEN. Consistent with the findings of Dasari et al., we observed increased incidence in the last 12 years compared to the previous (2). This has been observed with the increasing incidence of published reports on renal neuroendocrine neoplasms per decade, suggested to be due to the more widespread use of cross sectional imaging (4). In a review of published case reports of renal neuroendocrine neoplasms prior to 2006, Romero et al. observed half of patients to have distant metastatic disease. Similarly, most patients in our study had distant disease (42.2%), compared to local and regional disease (19.3 and 33.7%, respectively). This is dissimilar to other types of primary neuroendocrine tumors where local or regional disease is far more frequent than distant disease (1). Our data and those of Romero et al. suggest either a biphasic distribution of NETs versus NECs, or that the anatomic structure of renal NENs predisposes to early hematogenous metastatic spread.

We observed most tumors to be found in the renal parenchyma, with a minority (7.2%) in the renal pelvis. Others have observed similar location of tumor, despite no known native location of neuroendocrine cells in renal parenchyma. It is hypothesized that these tumors arise from neuroendocrine differentiation of pluripotent stem cells present in the parenchyma, misplaced neural crest cells in the kidney from embryogenesis, or development with congenital abnormalities of the kidney. We found a slightly greater frequency of tumors on the right side compared to the left, which has been observed more dramatically in other series (53.6% right) while a more recent series of literature reports found equal right and left sided tumors (10). The SEER database is limited by documenting laterality as right or left. As a consequence, we were unable to confirm previously published findings of increased risk of renal NET in horseshoe kidney (9).

In our study, slightly more than half of the patients were male, which is similar to prior observations. Our population had a median age of 59, which was slightly older compared to prior studies, where the median age was found to be 47, 49, and 52 (3, 4, 10).

The patients in our study were categorized in the SEER database by International Classification of Disease for Oncology, 3rd edition histology codes (ICD-O-3), localized to the kidney or renal pelvis, and required to be identified as the first and primary tumor for each patient across a broad time period. In our series, the earliest identified patient was in 1991. Over the last three decades, the terminology for neuroendocrine tumors has had significant changes. As an overall category, these tumors are NENs and are further divided into NET to include well-differentiated NET-G1 and NET-G2 (ICD-O 8240/3 and 8249/3 respectively), and neuroendocrine carcinoma, which would include NEC-NOS and LC-NEC (ICD-O 8246/3 and 8013/3 respectively).

There is no precise grading system for renal NENs, in part due to their rarity. While some descriptive histology features are correlated with poor prognosis, these features are inconsistently reported and are not recorded in the SEER database. Gastroenteropancreatic (GEP) NENs are graded on a basis of mitotic count and Ki-67 proliferation index, whereas pulmonary NENs are graded by mitotic count and extent of necrosis. The histologic data points of mitotic rate, Ki-67 proliferation index, and degree of necrosis are not discretely documented in the SEER database, which is a limitation of this population-based study.

The SEER database covers a broad timespan, and encompasses wide historical variance in classification styles. We have two possible explanations for the tumor category of NEC-NOS, based on the OS and DSS consistently being in-between that of NET-G1 and SC-NEC. It is possible that NEC-NOS represent what would now be called LC-NEC based on the IARC/WHO consensus proposal. Another possibility is that the NEC-NOS group is composed evenly of NETs (NET-G1 and NET-G2) and SC-NEC, which could represent an average OS and DSS. The paucity of NET-G2 cases, 2 total in 42 years of the database, suggests that NET-G2 is an underdefined category for renal NENs.

In our series, we looked at survival across various patient and tumor characteristics. We observed a 5-year OS of 50%. When SC-NEC was excluded, the 5-year OS rose to 62% with a median survival of 8.9 years. On multivariate analysis, more advanced stage was a predictor of poorer survival. Tumors classified as SC-NEC and NEC-NOS were associated with statistically significant hazard ratios of 7.22 and 2.35, respectively. This is consistent with observations in other organ systems that poorer survival is associated with NEC morphology and advanced stage (1). When sorted by tumor histology, the highest OS and DSS were seen in NET-G1 and the lowest in SC-NEC. Those with NEC-NOS showed OS and DSS in between NET-G1 and SC-NEC. Surprisingly, only two cases each were categorized as NET-G2 or LC-NEC, which may reflect historical terminology rather than the natural history of renal NENs. In this study, the paucity of NET-G2 and LC-NEC meant that survival data is underpowered.

Operative management remains the mainstay in curative treatment for NETs. In patients with local or regional disease, a majority underwent an operation. Only 2 of the 32 patients with local disease did not undergo resection and eventually died from pulmonary disease unrelated to the cancer diagnosis. In the patients with regional disease, 7 of 56 patients were not recommended to have an operation, and all died within 22 months of diagnosis. In patients with distant disease, OS and DSS were similar among those had an operation and those who did not. In this group, there may be limited long-term benefit to operative management, and other systemic treatment strategies may be more appropriate for these patients. In future studies, separating NET-G1 from all other categories of renal NENs will provide further insight as to the value of operative management.

Among NENs, those arising from the kidney are incredibly rare. For tumors limited to the kidney and adjacent retroperitoneum, surgery remains the key component to long-term survival for these patients. For patients with distant metastatic disease, systemic therapy may play a greater role in the management of these patients. We observed increased OS and DSS particularly in patients of younger age, earlier stage, and NET morphology. This study highlighted the limitations of studying an uncommon disease through terminology changes over time. Dedication to systematic classification and thorough data collection in SEER and other population databases will lead to more robust conclusions and understanding of these neoplasms. Integrating renal NENs into the larger international NEN dialogue and NEN databases will accelerate our knowledge for proper clinical management and accurate prognosis.

## Data Availability Statement

Publicly available datasets were analyzed in this study. This data can be found here: https://seer.cancer.gov/data-software/, Epidemiology, and End Results (SEER) database.

## Ethics Statement

Ethical review and approval was not required for the study on human participants in accordance with the local legislation and institutional requirements. Written informed consent from the participants’ legal guardian/next of kin was not required to participate in this study in accordance with the national legislation and the institutional requirements.

## Author Contributions 

AN: Conceptualization, methodology, formal analysis, investigation, visualization, writing—original draft. MO’L: Conceptualization, methodology, writing—review and editing. JA: Conceptualization, methodology, writing—review and editing. PI: Formal analysis, investigation, resources, data curation, writing—review and editing. JK: Conceptualization, supervision, writing—review and editing. DL: Conceptualization, supervision, writing—review and editing. GS: Conceptualization, supervision, writing—review and editing. SC: Conceptualization, visualization, supervision, writing—original draft, writing—review and editing. All authors contributed to the article and approved the submitted version.

## Conflict of Interest

The authors declare that the research was conducted in the absence of any commercial or financial relationships that could be construed as a potential conflict of interest.
